# Revealing cell–cell communication pathways with their spatially coupled gene programs

**DOI:** 10.1093/bib/bbae202

**Published:** 2024-05-05

**Authors:** Junchao Zhu, Hao Dai, Luonan Chen

**Affiliations:** Key Laboratory of Systems Biology, Shanghai Institute of Biochemistry and Cell Biology, Center for Excellence in Molecular Cell Science, Chinese Academy of Sciences, Cell building, No. 320 Yueyang Road, Xuhui District, Shanghai 200031, China; Key Laboratory of Systems Biology, Shanghai Institute of Biochemistry and Cell Biology, Center for Excellence in Molecular Cell Science, Chinese Academy of Sciences, Cell building, No. 320 Yueyang Road, Xuhui District, Shanghai 200031, China; Key Laboratory of Systems Biology, Shanghai Institute of Biochemistry and Cell Biology, Center for Excellence in Molecular Cell Science, Chinese Academy of Sciences, Cell building, No. 320 Yueyang Road, Xuhui District, Shanghai 200031, China; Key Laboratory of Systems Health Science of Zhejiang Province, School of Life Science, Hangzhou Institute for Advanced Study, University of Chinese Academy of Sciences, Chinese Academy of Sciences, No. 1, Xiangshan Zhinong, Xihu District, Hangzhou 310024, China

**Keywords:** cell–cell communication, spatial transcriptome, intercellular gene association, ligand–receptor pathway, spatial microenvironment

## Abstract

Inference of cell–cell communication (CCC) provides valuable information in understanding the mechanisms of many important life processes. With the rise of spatial transcriptomics in recent years, many methods have emerged to predict CCCs using spatial information of cells. However, most existing methods only describe CCCs based on ligand–receptor interactions, but lack the exploration of their upstream/downstream pathways. In this paper, we proposed a new method to infer CCCs, called Intercellular Gene Association Network (IGAN). Specifically, it is for the first time that we can estimate the gene associations/network between two specific single spatially adjacent cells. By using the IGAN method, we can not only infer CCCs in an accurate manner, but also explore the upstream/downstream pathways of ligands/receptors from the network perspective, which are actually exhibited as a new panoramic cell-interaction-pathway graph, and thus provide extensive information for the regulatory mechanisms behind CCCs. In addition, IGAN can measure the CCC activity at single cell/spot resolution, and help to discover the CCC spatial heterogeneity. Interestingly, we found that CCC patterns from IGAN are highly consistent with the spatial microenvironment patterns for each cell type, which further indicated the accuracy of our method. Analyses on several public datasets validated the advantages of IGAN.

## Graphical Abstract

**Graphical Abstract:**
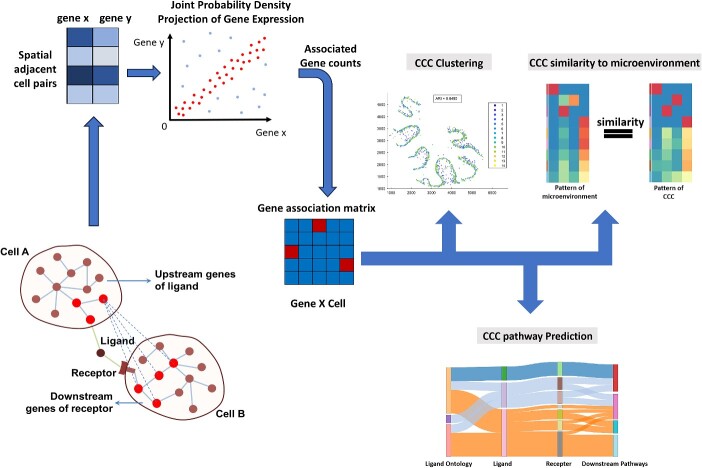

## INTRODUCTION

Many methods have been developed to infer cell–cell communication (CCC) from scRNA-seq data, such as CellPhoneDB [1], CellChat [2] and NicheNet [3]. These methods estimate the possibility of CCC between two cell types based on the expression levels of known ligand–receptor pairs or downstream genes of receptors. As the communications between cells are often related to their spatial distance, a number of methods have emerged to predict CCC by using spatial information of cells, with the rise of spatial transcriptomics in recent years. Some methods use spatial proximity as a constraint for CCC. Giotto [4] and stLearn [5] just calculate the pairs of adjacent cells in space; SpaTalk [6] uses sorting to test the number of co-expressed counts of ligands and receptors in spatial neighborhoods; COMMOT [7] and spaOTsc [8] predict spatial CCC through optimal transmission algorithms, where the loss of ‘ligand–receptor transport’ between long-distance cells is set to be large. Other methods use neighborhood relationships between cells as inputs to predict CCC. GCNG [9] uses graph convolutional neural networks to integrate gene expression data and spatial neighbor networks; DeepLinc [10] uses graph autoencoders for encoding and decoding reconstruction of spatial neighbor networks. Although these methods have their advantages in predicting CCC, most existing methods only describe CCC based on ligand-receptor interactions, but lack the exploration of their upstream/downstream pathways. In fact, production of ligands needs the cooperation of many upstream genes, and effect of receptors needs the cascade reaction of downstream genes. If two cells are interacted with each other, a wide range of genes will be associated, including the upstream genes of ligands and downstream genes of receptors. Thus, if we describe CCC from a network viewpoint, we can not only infer CCC in an accurate manner, but also discover extensive information for the regulatory mechanisms behind CCC.

In this work, we developed a new method called Intercellular Gene Association Network (IGAN). By IGAN method, it is for the first time that we can estimate the intercellular gene-associations between two single spatially adjacent cells, i.e. the association between gene *x* of cell *A* and gene *y* of cell *B*, where cell *A* and cell *B* are spatially adjacent based on spatial transcriptomic data. This method assumes that the genes involved in CCC have stronger intercellular gene-associations. Even if the expression of ligand or receptor is low, relevant CCC can still be detected. In addition, IGAN can measure the CCC activity at single cell/spot resolution, and help to discover the CCC spatial heterogeneity. Interestingly, we found that CCC patterns from IGAN are highly consistent with the spatial microenvironment patterns for each cell type, which further validated the accuracy of our method and can be used to reveal the microenvironment specific CCC.

## MATERIAL AND METHODS

### Overview of IGAN

The interaction between cells often leads to changes in gene expression [11–13], causing fluctuations in gene expression within the same cell type and exhibiting associations of gene expression in interacting cells.

**Figure 1:**
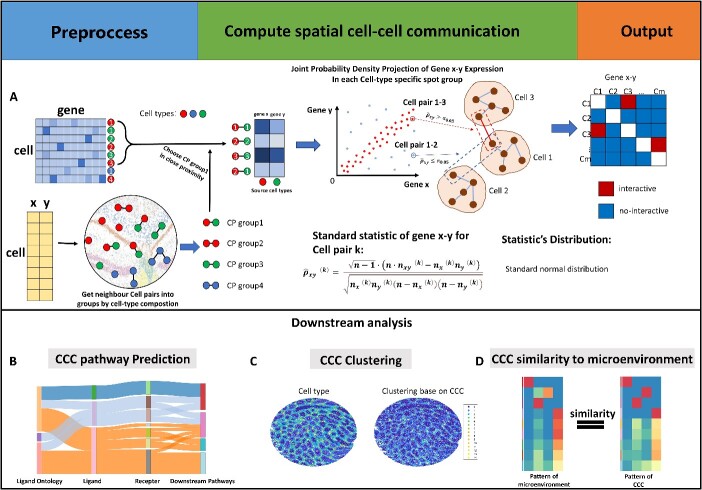
Overview of IGAN. (**A**) The workflow of IGAN. IGAN infer CCC based on cell-pair gene–gene associations. (**B**) Panoramic cell-interaction-pathway graph (Sankey graph) of pathway-based cell–cell communication analysis. (**C**) Clustering based on CCC information produced by IGAN. (**D**) Similarity between CCC patterns and spatial microenvironment patterns for each cell type.

In this work, we proposed IGAN to identify the intercellular gene associations. To perform IGAN, we require spatial transcriptomic data as input, including a gene expression matrix and cell coordinates (Figure 1A). Within each cell pair, we perform a non-parametric statistical model to construct the cell–cell gene association network. This statistical model is based on the necessary and sufficient condition of statistical independence, and a statistic is designed for hypothesis test to determine whether two genes are associated across cells. This statistic is calculated based on the approximation of joint probability density and marginal probability densities of two genes.

By IGAN method, we can perform three types of unsupervised downstream analyses besides ligand–receptor interactions.

1. IGAN can perform pathway-based CCC analysis to construct a new panoramic cell-interaction-pathway graph (Sankey graph of cell-interaction-pathways), thus enabling the discovery of related pathways involving ligand–receptor interactions and helping biologists understand the pathway mechanisms behind CCC (Figure 1B).
2. IGAN can cluster cells/spots based on their CCC patterns, thus identifying heterogeneity from the perspective of intercellular communication (Figure 1C).
3. Based on the consistency between CCC patterns and microenvironment patterns of each cell type, we can quantify the accuracy of the CCC inferred by IGAN and other methods. We can also reveal the microenvironment specific CCC by comparing the similarities between CCC patterns and microenvironment patterns. (Figure 1D).

### Identification of intercellular gene-associations

The key issue of IGAN is how to identify each gene-pair association across cells. In this study, we first make two cells (or spots) *a_k_* and *b_k_* to form a cell pair *a_k_b_k_*. The two cells exhibit short Euclidean distances, as determined by their spatial transcriptome coordinates. To define the threshold distance, we first calculated the nearest distances calculated between each cell and all other cells. Subsequently, considering the presence of outlier cells, the 99th percentile of the nearest distances was chosen. If the data is from 10×, threshold distance is defined as the minimum distance between all cell pairs. In cell pair *a_k_b_k_*, Cell *a_k_* belongs to cell type A and *b_k_* belongs to cell type B. If A and B are not equal, *a_k_* and *b_k_* correspond to different cell types, while if A is equal to B, *a_k_* and *b_k_* correspond to the same cell type. Then we select *n* cell pairs *a*_1_*b*_1_, *a*_2_*b*_2_, …, *a_n_b_n_*, in which *a*_1_, *a*_2_, …, *a_n_* belong to cell type A and *b*_1_, *b*_2_, …, *b_n_* belong to cell type B. Next, we make a scatter diagram based on the *n* cell pairs where plot *k* represents cell pair *a_k_b_k_* (Figure S1). X-axis represents the expression of gene *x* in *a*_1_, *a*_2_, …, *a_n_*, and Y-axis represents the expression of gene *y* in *b*_1_, *b*_2_, …, *b_n_*. We extended the previously proposed cell-specific network (CSN) model [14], and developed a new method with non-parametric statistical testing (Supplementary Note 1 and Figures S1 and S2).

### Pathway analysis of IGAN

Before the statistic test, we should first normalize the spatial transcriptome data by R package Seurat [15], and then pre-cluster all cells to several clusters. Then we should calculate the adjacent cells for each cell in space, based on the spatial coordinate of spatial transcriptome.

For cell *a*, if there are *t_a_* adjacent cells ${b}_1\dots{b}_{t_a}$, we can make *t_a_* cell pairs ${ab}_1\dots a{b}_{t_a}$, and construct *t_a_* intercellular gene association networks ${H}^{\left({ab}_1\right)}\dots{H}^{\left({ab}_{t_a}\right)}$, where ${H_{ij}}^{\left({ab}_k\right)}$ represents the association (1 or 0) between gene *i* of cell *a* and gene *j* of cell *b_k_* (*k =* 1*, …, t_a_*). Note that ${H}^{\left({ab}_k\right)}$ is not a symmetric matrix. If gene *i* of cell *a* is associated with many genes of cell *b_k_*, representing the importance of gene *i* in CCC, $\sum_{j=1}^m{H_{ij}}^{\left({ab}_k\right)}$ will be a large value (*m* is the number of genes).

We can combine our IGAN method with known ligand–receptor interactions which is from cellchat [2] and exhibit CCC as a new panoramic cell-interaction-pathway graph. In our method, we describe the strength of CCC through the association counts between the ligands and the downstream genes of receptors. First, we use ligand gene *i* of $\frac{\sum_{k=1}^{t_a}\sum_{j=1}^m{H_{ij}}^{\left({ab}_k\right)}}{t_a}$ to represent the sending signal from cell *a*, similarly using ligand gene i, the intensity of signal reception is defined as the sum of the influences of ligand i from neighboring cells on cell a. $\frac{\sum_{k=1}^{t_a}\sum_{j=1}^m{H_{ij}}^{\left({b}_ka\right)}}{t_a}$ represents the receipting signal to cell *a*, and use $\frac{\sum_{k=1}^{t_a}\sum_{j=1}^m{H_{ij}}^{\left({ab}_k\right)}}{t_a}+\frac{\sum_{k=1}^{t_a}\sum_{j=1}^m{H_{ij}}^{\left({b}_ka\right)}}{t_a}$ to measure the CCC activity of ligand gene *i* in cell *a*. Thus, single cell/spot *a*’s overall CCC activity can be represented by sum of all ligand genes’ CCC activity: 


(1) \begin{align*} CCC\ strength=\sum_{i=1}^l\left(\frac{\sum_{k=1}^{t_a}\sum_{j=1}^m{H_{ij}}^{\left({ab}_k\right)}}{t_a}+\frac{\sum_{k=1}^{t_a}\sum_{j=1}^m{H_{ij}}^{\left({b}_ka\right)}}{t_a}\right) \end{align*}

where *l* is the number of ligand genes.

Next, we selected ligands with high CCC activity and identified their downstream genes that exhibited an association of >0.01*N counts with the corresponding ligand among all cell pairs, where *N* represents the total number of cell pairs. Subsequently, KEGG enrichment analysis was conducted on the downstream genes. Enriched pathways would be chosen if they contained the receptors that correspond to the selected high CCC activity ligands. Finally, we performed GO/KEGG enrichment analysis on the ligands with high CCC activity to obtain their upstream functions. All above this enables us to explore how ligands exert their biological functions of GO by influencing the downstream pathways of neighboring cells. Thus, we inferred a panoramic cell-interaction-pathway graph consisting of ligand upstream functions, ligands, receptors, and downstream pathways.

### Clustering based on cell–cell communication features

As mentioned above, the strength of CCC is the association counts between the ligands and the downstream genes of receptors. We can use the sending signal and receipting signal to describe the CCC features of each single cell. If there are *n* cells and *p* ligand genes, for cell a and ligand i, the ‘sending feature’ is the average count of target genes correlated with ligand i in neighboring cells, whereas the ‘receiving feature’ is the average count of target genes in cell a that are correlated with neighboring cells’ ligand i. we can construct a matrix *F* with *n × 2p* elements, which is 


(2) \begin{align*} {F}_{ai}=\left\{\begin{array}{@{}l}\dfrac{\sum_{k=1}^{t_a}\sum_{j=1}^m{H_{ij}}^{\left({ab}_k\right)}}{t_a}\kern4.5em i=1,\dots, p\\[8pt] {}\dfrac{\sum_{k=1}^{t_a}\sum_{j=1}^m{H_{i-p,j}}^{\left({b}_ka\right)}}{t_a}\kern3.5em i=p+1,\dots, 2p\end{array}\right. \end{align*}

We can use the Seurat package [15] to cluster normalized *F* matrix and obtained the cell clustering. The details are in Supplementary Note 6.

### Method comparison

Here we defined the ‘CCC pattern’ as the combination of CCCS mediated by different types of ligands, and the ‘microenvironment pattern’ as the combination of various types of microenvironments. The CCC pattern of each cell is found to be consistent with its microenvironment pattern, and thus we can compare the performance of different CCC inference methods based on this consistency.

First, we use the recently published method Spatial Omics mulTIPle-task analysis (SOTIP) [16] to obtain the spatial microenvironment matrix ${E}_{m\times n}$, where *m* is the number of cell types in the dataset, and *n* is the number of microenvironment types calculated by SOTIP. *E_ij_* represents the number of cells belonging to the microenvironment *j* in the cell type *i*. Subsequently, we use the cellchat package to perform non-negative matrix factorization on matrix *E*, finding the most suitable number of CCC patterns *k*, and obtaining the decomposed *m* × *k* matrix *E^a^* and *k* × *n* matrix *E^b^*. ${E}_{ij}^a$ represents the contribution of cell type *i* to pattern *j*, and ${E}_{ij}^b$ represents the contribution of microenvironment type *j* in the pattern *i*.

Based on the CCC calculation results, we construct the cell communication matrix ${C}_{m\times l}$, where *m* is the number of cell types in the dataset, and *l* is the number of ligands. ${C}_{ij}$ represents the influence of ligand *j* in the cell type *i*, and can be calculated as eqn. (8). 


(3) \begin{align*} {C}_{ij}=\left\{\begin{array}{@{}l}\displaystyle \sum_{i^{\prime}\ne i}^m\frac{\sum_{a\in i,b\in{i}^{\prime}}^{N^{i{i}^{\prime }}}{H}_{j\cdot}^{(ab)}}{N^{i{i}^{\prime }}}\kern3.4em sending\ signal\\{}\displaystyle \sum_{i^{\prime}\ne i}^m\frac{\sum_{a\in i,b\in{i}^{\prime}}^{N^{i{i}^{\prime }}}{H}_{j\cdot}^{(ba)}}{N^{i{i}^{\prime }}}\kern3.25em receipting\ signal\end{array}\right. \end{align*}

Where ${N}^{i{i}^{\prime }}$ is sum counts of cell pairs which is composed of cell type $i$ and ${i}^{\prime }$. Actually, we get 2 cell communication matrix *C* which represent sending and receipting signal respectively. Subsequently, we also utilized the cellchat package [2] to perform non-negative matrix factorization on matrix *C*, setting the number of CCC patterns as *k*, which is consistent with the decomposition of microenvironment matrix *E*, and obtaining the decomposed matrix ${C}_{m\times k}^a$ and ${C}_{k\times l}^b$. ${C}_{ij}^a$ represents the contribution of cell type *i* to the CCC pattern *j* and ${C}_{ij}^b$ represents the contribution of gene *j* in pattern *i*.

We measure the quality of our method by comparing the similarity between ${E}_{m\times k}^a$ and ${C}_{m\times k}^a$, which describe the pattern of microenvironment and CCC respectively. Here, we use weighted KL divergence to measure the similarity of the two matrixes. 


(4) \begin{align*} & {KL}_w=\sum_{i=1}^m\sum_{j=1}^k{W}_{celltype}^i \cdot{W}_{pattern}^j \cdot{E}_{ij}^a \cdot \log \frac{E_{ij}^a}{C_{ij}^a} \nonumber \\& {W}_{celltype}^i=\frac{N_i}{\sum_{i=1}^m{N}_i} \qquad{W}_{pattern}^j=\sum_{z=1}^n{E}_{jz}^b \end{align*}

where ${N}_i$ is the number of cells belonging to cell type *i*. ${W}_{celltype}^i$ is the proportion of cell type *i* to all cells, higher proportion means more important of this cell type. ${W}_{pattern}^j$ is the contribution of all microenvironment types calculated by SOTIP to pattern *j*, larger ${W}_{pattern}^j$ means more important of pattern *j*. At last, the smaller ${KL}_w$ means the more similar between microenvironment and CCC patterns, and means the better performance of CCC inference method.

## RESULT

### Revealing CCC upstream/downstream pathways

**Figure 2:**
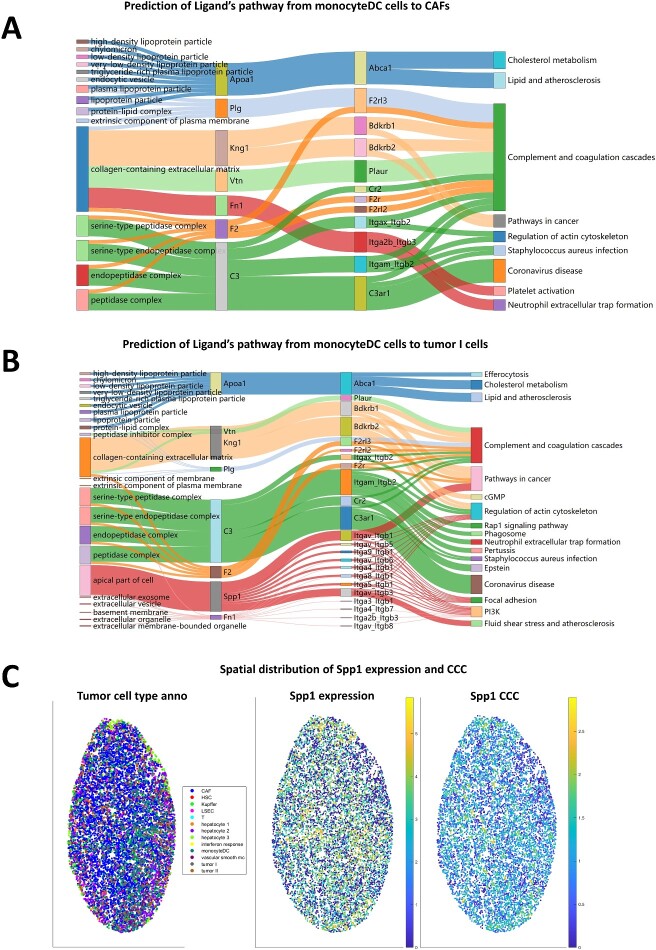
Revealing CCC upstream/downstream pathways in the liver cancer dataset. (**A**) Sankey graph of cell interaction pathways in the cancer dataset from monocyteDC cells to CAFs. The first column represents Ligand Ontology, while the second column represents high-activity ligands. The third column represents the receptors corresponding to the ligands in the cellchatDB. The fourth column represents the predicted downstream pathways affected by these ligands. The thickness of the flow from the third column to the fourth column represents the number of downstream target genes in the pathway which is associated with the ligand. The thicker the flow, the greater the impact of the ligand-receptor pair on the corresponding downstream pathway. (**B**) Sankey graph of cell interaction pathways in the cancer dataset from monocyteDC cells to Tumor I cells. (**C**) Spatial distribution of Spp1 expression (middle) and the number of associated genes in the adjacent cells (right). The left is the cell-type overview of the dataset.

We performed our IGAN method on a liver cancer dataset [17]. This spatial transcriptome dataset is sequenced by Slide-seq. By using our pathway-based CCC method, as shown in Figure 2A, we find seven ligands, Apoa1, Plg, Kng1, Vtn, Fn1, F2, C3 (Supplementary Note 3 and Figures S4 and S5), are highly active in CCC from monocyteDC cells to CAFs, and enriched nine downstream pathways that may be affected by these ligands. A shown in Figure 2B, we find 8 ligands, Apoa1, Vtn, Kng1, Plg, C3, F2, Spp1 and Fn1 (Supplementary Note 3 and Figs S4 and S5), are highly active in CCC from monocyteDC cells to Tumor I cells, and enriched 17 downstream pathways that may be affected by these ligands. Figure 2A and, B illustrate that monocyte DC exhibit varying impacts on CCC when interacting with different target cell types, indicating that variations in the cellular microenvironment can lead to distinct CCC outcomes. Moreover, many downstream pathways are simultaneously influenced by multiple ligands, indicating that multiple ligands synergistically regulate these pathways. For example, as shown in Figure 2B, ‘Pathways in cancer’ is simultaneously affected by three active ligands, among which Spp1 ligand influences it through eight receptor, KNG1 influences it through Bdkrb1 and Bdkrb2 receptors and C3 influences it through Itgax_Itgb2 and Itgam_Itgb2.

Figure 2B shows that Spp1 is associated with many downstream genes and affects many pathways. Previous studies have indicated that Spp1 plays an important role in liver cancer [18]. SPP1 can influence the immune regulation [19], cell survival [19], tumor progression [19, 20] and prognosis [21] by affecting the tumor immunological microenvironment [21]. Figure 2C illustrates the spatial distribution of SPP1, both the gene expression and the number of associated genes in the adjacent cells (after logarithm). We can see that the high expression area highly overlaps with the high association area.

### Identifying spatial clusters by cell clustering with cell-pair gene-associations/networks

**Figure 3:**
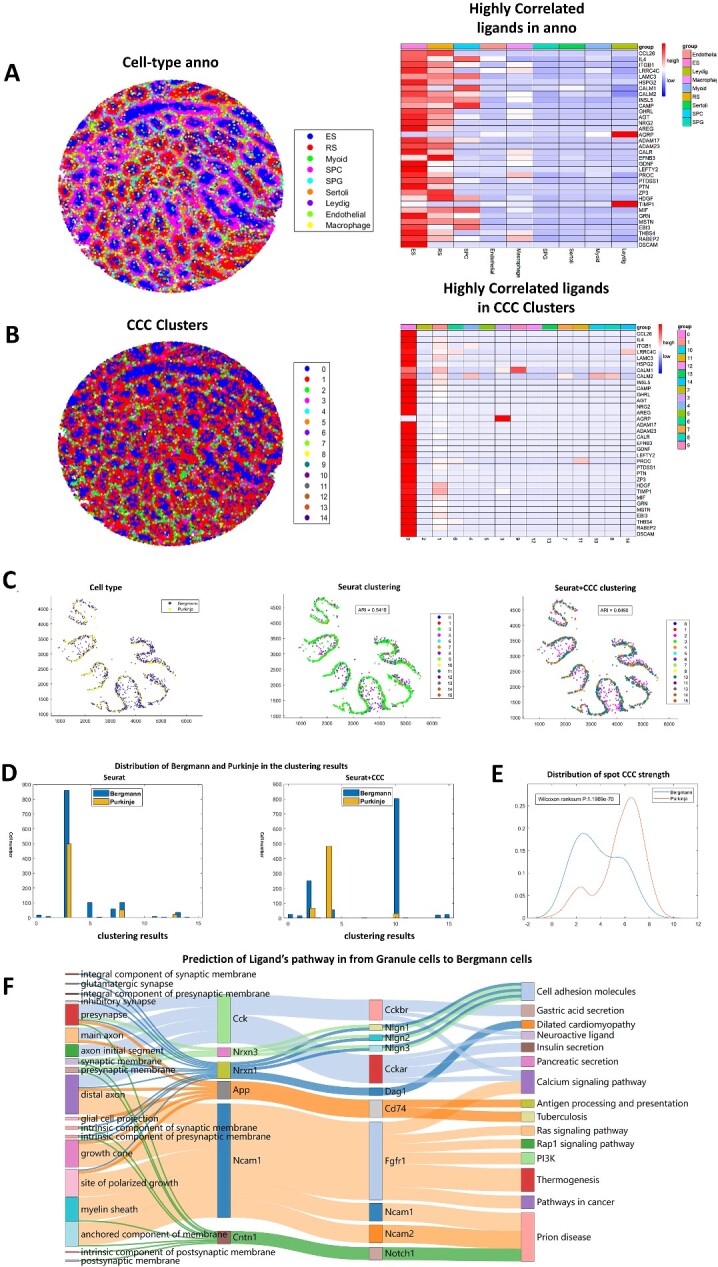
Identifying spatial clusters by cell clustering with CCC intercellular gene-associations in IGAN. (**A**) Cell-type overview of the mouse testes dataset (left) and heatmap of the expression levels of high-activity ligands in annotated cell types. (**B**) Overview of clustering result based on CCC information (left) and heatmap of the expression levels of high-activity ligands in CCC clusters. (**C**) Spatial overview of Purkinje and Bergmann cells annotated by RCTD (left). Spatial overview of clustering results by Seurat [15], ARI = 0.5419 (middle). Spatial overview of clustering results by Seurat [15] with added CCC information, ARI = 0.6490 (right). (**D**) Distribution of Purkinje and Bergmann cells in the Seurat [15] clustering result (left) and Seurat [15] clustering result with added CCC information (right). The X-axis represents the clustering categories and the Y-axis represents the number of Purkinje and Bergmann cells in each clustering categories. (**E**) Distribution of CCC strength in Purkinje and Bergmann cells. CCC strength of Purkinje cells is significantly higher than Bergmann cells, *P* = 1.1969e-70. (**F**) Sankey graph of cell interaction pathways in the cerebellum dataset from Purkinje cells to Bergmann cells.

We performed cell clustering based on CCC information in a dataset of mouse spermatogenic tubules [22]. We find that the CCC is mainly active in elongating/elongated spermatid (ES) and round spermatid (RS) (Figure 3A). But from the result of CCC clustering (Figure 3B), we find that ES and RS are classified into the same cluster (Supplementary Note 4 and Table S1), which indicates that the CCC patterns of ES and RS are similar.

On the other hand, we may find new cell heterogeneity from the perspective of CCC. In a dataset of human cerebellum [23], we can separate Purkinje cells and Bergmann cells after adding the information of CCC in cell clustering. These two cell types mix together in space and locate between the Molecular layer and Granular layer. Traditional computational methods cannot distinguish the two cell types (Figure 3C), which are annotated by experimental methods. As shown in Figure 3D, the two cell types are both classified into Cluster 3 by Seurat [15], but after adding CCC information calculated by IGAN based on Seurat clustering result, Bergmann cells is classified into Cluster 3 and Cluster 10, and Purkinje cells is classified into Cluster 4. Furthermore, we calculate the number of associated genes as CCC strength, and find that the distribution of CCC strength is significantly different (*P* = 1.1969e-70, Wilcoxon rank sum test) between Purkinje cell and Bergmann cell (Figure 3E), which further indicates the effect of CCC information in distinguishing the two cell types. It should be noted that if we only use CCC information and exclude the expression data in cell clustering, we will identify CCC patterns instead of cell types, and the cells on the border of two cell types trend to cluster together (Supplementary Note 5 and Figure S6).

We also performed our pathway-based method on this cerebellum dataset from Granule cells to Bergmann cells. Figure 3F illustrates the six ligands (Figure S7) and 11 receptors with the highest activity, and their associated pathways. Ncam1 is a ligand that can regulate the biological processes including neuroinflammation [24], neuron migration [25], synaptic formation [26, 27] and signal transduction [28]. Our method finds that it acts on the several pathways through Fgfr1, Ncam1 itself and Ncam2. In addition, notch signal plays an important role in nervous system [29]. Our result found that Cntn1 influences Prion disease pathway through Notch1 receptor.

### Elucidating spatial heterogeneity of CCC activity at single cell/spot level

**Figure 4:**
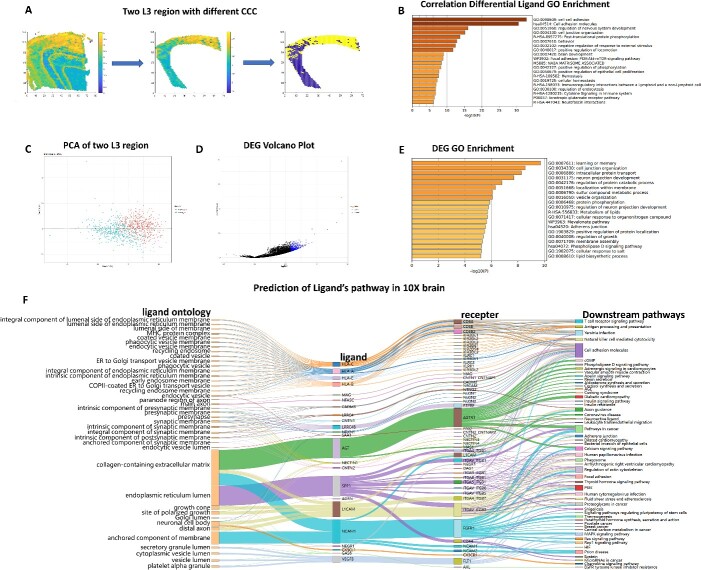
CCC spatial heterogeneity at single cell/spot level in the 10X brain dataset. (**A**) CCC strength overview of the brain dataset (left). CCC strength overview of the L3 layer (middle). L3 layer is divided into two regions. (**B**) GO analysis of the 88 significantly different ligands. (**C**) PCA of two L3 regions. (**D**) DEG Volcano Plot of high CCC strength L3 region and low CCC strength L3 region. (**E**) GO analysis of the DEGs. F. Sankey graph of cell interaction pathways in the 10X brain dataset.

We also performed our method on a human brain dataset [30] that was sequenced by 10X spatial transcriptome. In Figure 4A, we measured the CCC strength at single spot level, which was defined by eqn. (6). We can see that the border between two cell types usually has high CCC strength. In addition, we note that the spatial distribution of CCC strength within the L3 cortex annotated by the dataset is not uniform. We can divide the L3 cortex into high CCC strength region (yellow area) and low CCC strength region (blue area). We further analyze the difference between the two regions, and find the sending signals of 88 ligands are significantly different. GO analysis by metascape website of these 88 ligands shows the significant enrichment of cell-adhesion-related pathways (Figure 4B). Neural cell adhesion molecules 1 (NCAM1) is one of the 88 ligand we found. NCAM1 is glycoprotein that mediates the interactions between two cells or between cell and extracellular matrix. It plays a certain role in neuroinflammation [24], nerve regeneration [26, 27], transmembrane signal transduction [28], learning and memory [25] and so on. In addition, we also enriched the pathways of ‘regulation of nervous system development’, ‘cell junction organization’, ‘ionotropic glutamate receptor pathway’ and ‘neurofascin interactions’, which are all related to the function of nervous system. These biological functions are more active in L3 cortex with high CCC strength regions than in low CCC strength regions.

Next, to further analyze the difference between the two regions, we performed PCA on the spots of L3 cortex. We find that the spots of the two regions are obviously clustered into two parts (Figure 4C). Subsequently, we use edgeR package [31] to perform the differential expression analysis, and find a total of 492 differential expression genes (DEGs), most of which are upregulated in high CCC strength regions (Figure 4D). GO/KEGG enrichment analysis indicates that these DEGs are mainly enriched to cell-adhesion-related pathways such as ‘focal adhesion’, ‘phospholipase D signaling pathway’, ‘cell adhesion molecules’, and are also enriched to the neural-system-related functions such as ‘learning or memory’, ‘cell junction organization’, and ‘neuron projection development’ (Figure 4E). Both DEGs and ligands are enriched to cell-adhesion-related functions, indicating the stronger adhesion of neurons in high CCC strength region.

Finally, we also performed pathway-based CCC analysis on this 10X dataset. We illustrate 23 ligands (Supplementary Note 6 and Figures S8 and S9), and their associated pathways (Figure 4F). We find that SAA1 and AGT jointly affect the ‘neuroactive ligand pathway’. In addition, the ‘axon guidance pathway’, which is one of the key steps in the formation and functional development of nervous system [32], is jointly regulated by four ligands: LRRC4, CNTN2, L1CAM and NCAM1. Among them, ligand LRRC4 acts on receptor NTNG2, while ligands CNTN2, NCAM1 and L1CAM all act on receptor L1CAM.

### Comparison of CCC patterns and microenvironment patterns

**Figure 5:**
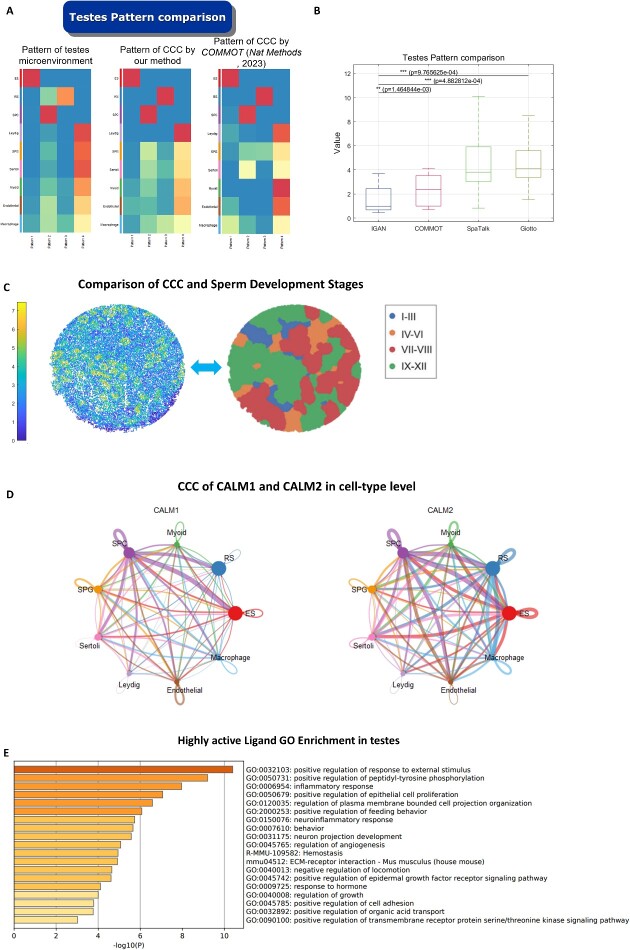
Comparison of CCC patterns and microenvironment patterns in the mouse seminiferous tubule datasets. (**A**) Microenvironment pattern and CCC pattern. The left is microenvironment pattern of WT3 dataset. The middle is CCC pattern of WT3 dataset based on our method IGAN. The right is CCC pattern of WT3 dataset based on COMMOT. (**B**) Comparison of the consistency between the CCC pattern and the microenvironment pattern in IGAN and COMMOT methods. The 12 data point represent 6 sending signal CCC pattern and 6 receiving signal pattern. As the value decreases, there is a greater resemblance between the microenvironment pattern and CCC pattern. The Wilcoxon test *P* = 0.0015. (**C**) Left: CCC activity overview of WT1 dataset. Right: sperm development stages [22]. (**D**) CCC of CALM1 and CALM2 in cell-type level. The thickness of the flow represents the strength of the interactions. (**E**) GO analysis of the high activity ligand in WT3 dataset.

We assume that CCC of each cell is associated with its microenvironment. By comparing the consistency of CCC patterns and microenvironment patterns for each cell type, we can validate the accuracy of CCC inference methods. In this work, we used a set of mouse seminiferous tubule datasets [22] including three datasets of wild type and three datasets of diabetes type. For each dataset, we performed non-negative matrix factorization by cellchat package to obtain the spatial microenvironment pattern from SOTIP method, CCC pattern from our method, and CCC pattern from the COMMunication analysis by Optimal Transport (COMMOT) method (Figure 5A). Obviously, CCC patterns from IGAN are quite similar to the microenvironment patterns, which validate the accuracy of IGAN. Then, we compared the consistency between the spatial microenvironment pattern and CCC pattern between different methods (our method compared with COMMOT, SpaTalk and Giotto). The result shows that the KL-divergence between CCC of our method and spatial microenvironment is significantly smaller than that of other methods, showing the better performance of our method (Figure 5B).

Interestingly, we found that CCC patterns of a specific cell type have a small KL-divergence with its spatial microenvironment patterns (Figure 5B), which means that they are highly consistent or associated and may be further used to reveal the microenvironment-pattern-specific CCC. For example, in WT3 dataset, the microenvironment pattern 4 mainly represents cell type Leydig, Sertoli and SPG (Supplementary Note 7 and Figure S10). In its corresponding CCC pattern 4, it is mainly featured by ligand TIMP1, CD80 and AGRP (Figure S11).

We performed further analysis on one of the six datasets, WT1, in which the cells were divided into four stages during sperm development [22]. Our method reveals that in the IX-XII stage, the CCC activity of sperm cells is significantly higher than other stages (Figure 5C), which indicates that CCC plays an important role in the final stage of sperm development. In addition, during the various stages of spermatogenesis, the major cell types of sperm cells are different, with their developmental sequence being as follows: SPG - > SPC - > RS - > ES, where ES is the major cell type in the last stage. Thus, we employed the rank sum test to compare the CCC activity among the above four types of cells, and also revealed that ES cells exhibit the highest level of CCC activity. This finding is consistent with the results presented in Figure 5C and we got the same results in five other datasets. All these results have been added to Supplementary Note7. Moreover, we find that calmodulin has high activity in the CCC (Figure 5D), suggesting the important role of calcium ions in sperm development [33]. At last, we perform GO analysis of the high activity ligand in WT3 dataset (Figure 5E) by metascape website and find many important pathways related to sperm development including ‘positive regulation of peptidyl-tyrosine phosphorylation’, ‘positive regulation of response to external stimulus’, ‘positive regulation of epithelial cell proliferation’ and ‘regulation of plasma membrane bounded cell projection organization’.

## DISCUSSION

A biological process can be seen as a dynamic process with gene expressions as variables [34, 35]. Even in a single cell, the gene expression is always changing with time and cell state, which results in the difficulty to quantify cell heterogeneity and CCC. On the other hand, the correlation between genes is relatively stable or less variable. In this study, we developed a new statistical test model IGAN, which changes the gene expression into joint probability to calculate the associations between genes. Based on the more stable associations, theoretically we can obtain more reliable inference of CCC.

In CCC, ligand–receptor pairs are crucial as mediators, and many CCC inference methods used known ligand–receptor interactions as a starting point. However, ligand–receptor interactions are actually protein interactions, which cannot be directly described by gene expression data. The production of ligands involves the coexpression of a series of upstream genes, these ligands act on other cells through receptors, and result in the expressed changes of many downstream genes. Thus, the expressions of many genes are associated with each other across cells, and we can use pathway–pathway interactions to describe CCC. In fact, understanding CCC from the perspective of pathways has of great significance for elucidating biological mechanisms. Our method can identify the associated gene pairs between two single cells or two spots from spatial transcriptome data. The analysis of CCC is no longer limited to ligand–receptor interactions, but extends to the associations of all genes. Thus, we can characterize CCC from spatial gene associations and calculate CCC activity as the sum of the associated gene pairs. Then, based on the pathway enrichment of all associated genes, the ligands and receptors can be directly identified from the enriched pathways, and exhibited as a panoramic cell-interaction-pathway graph, including some complicated biological processes such as synergism of multiple ligands. In addition, our method can measure the CCC features at single cell or single spot level. Thus new cell heterogeneity may be found from the CCC features, including different cell types or different cell states.

In the CCC analysis, due to the incomplete information known in biology and the limitation of the simulated datasets, evaluating the accuracy of CCC results is always a challenge. In this study, we find that CCC is associated or consistent with the microenvironment in which the cells are located. Even for the same cell type, cells in different microenvironments may have different CCC patterns. By comparing the consistency between CCC patterns and microenvironment patterns, we can evaluate the accuracy of the discovered CCC. This is an unsupervised scheme, and a higher consistency score means that the result of CCC estimation is more accurate.

**Key Points**

- A statistical testing framework was constructed to estimate the intercellular gene associations.
- A novel method was presented to infer upstream/downstream pathways of cell–cell communications from the network perspective.
- A general benchmark method was proposed to evaluate the accuracy of spatial cell–cell communication recognition methods, utilizing the spatial microenvironment patterns.

## CODE AVAILABILITY

The source code is available in GitHub repository (https://github.com/Zhu-JC/IGAN).

## Supplementary Material

Supplemental_Data_bbae202

## Data Availability

All the datasets used in this study are publicly available and accessible: The slide.seqV2 liver cancer dataset is available at the Broad Institute Single Cell Portal (https://singlecell.broadinstitute.org/single_cell/study/SCP1663), file’s name is “SpatialRNA_cropped_slideseq_tumor.rds.zip”. It is obtained from KrasG12D/+ Trp53-/- (KP) mouse tumor model and includes 7653 cells and 13 celltypes (CAF, HSC, Kupffer, LSEC, T, hepatocyte 1, hepatocyte 2, hepatocyte 3, interferon response, monocyte_DC, vascular smooth mc, tumor I, tumor II). Every cell has 21902 genes detected. The slide.seqV2 cerebellum dataset is available at the Broad Institute Single Cell Portal (https://singlecell.broadinstitute.org/single_cell/study/SCP948), files’ name are “Cerebellum_BeadLocationsForR.csv” and “Cerebellum_MappedDGEForR.csv”. It is collected on the adult mouse cerebellum as the target and includes 39496 cells and 19 cell types (Astrocytes, Bergmann, Candelabrum, Choroid, Endothelial, Ependymal, Fibroblast, Globular, Golgi, Granule, Lugaro, MLI1, MLI2, Macrophages, Microglia, Oligodendrocytes, Polydendrocytes, Purkinje, UBCs). Every cell has 23096 genes detected. The slide.seqV2 mouse testes datasets are available in the dropbox (https://www.dropbox.com/s/ygzpj0d0oh67br0/Testis_Slideseq_Data.zip?dl=0). It includes 3 WT mouse testes datasets and 3 DB mouse testes datasets. It is obtained from adult male mice of 3–10-month-old and includes 9 celltypes(ES, RS, Myoid, SPC, SPG, Sertoli, Leydig, Endothelial, Macrophage). The seminiferous tubules in these 6 datasets are divided into four stages, representing the four periods of sperm development. The 10X 151673 DLPFC Human Brain Layers dataset is available through LIBD (http://research.libd.org/spatialLIBD/). It is obtained from the human DLPFC that spans six neuronal layers plus white matter and includes 3639 cells with 24841 genes. The ligand and target genes’ name is produced by NichNet11 which is available at Zenodo (https://doi.org/10.5281/zenodo.3260758).

## Author Biographies

**Junchao Zhu** is a PhD student in Center for Excellence in Molecular Cell Science. His research interest is bioinformatics and deep learning.

**Hao Dai** is a associate research fellow in Center for Excellence in Molecular Cell Science. His research interest is bioinformatics and network biology.

**Luonan Chen** is a professor and executive director at Key Laboratory of Systems Biology in Center for Excellence in Molecular Cell Science. His interests include systems biology, computational biology and applied mathematics.
