# Supplementary material for: Revealing cell–cell communication pathways with their spatially coupled gene programs
Source: Brief Bioinform. 2024 May 5;25(3):bbae202. doi: 10.1093/bib/bbae202 (PMC11070651; doi:10.1093/bib/bbae202)
Supplement: Supplemental_Data_bbae202 [file supplemental_data_bbae202.pdf]

Inferring cell-cell communication pathways by spatial gene networks based on spatial transcriptomic data

Supplementary Data

Junchao Zhu[1], Hao Dai[1,*], Luonan Chen[1,2,*]

[1] *Key Laboratory of Systems Biology, Shanghai Institute of Biochemistry and Cell Biology, Center for Excellence in Molecular Cell Science, Chinese Academy of Sciences, Shanghai 200031, China.*

[2] *Key Laboratory of Systems Health Science of Zhejiang Province, School of Life Science, Hangzhou Institute for Advanced Study, University of Chinese Academy of Sciences, Chinese Academy of Sciences, Hangzhou 310024, China.*

* To whom correspondence may be addressed: Luonan Chen: lnchen@sibs.ac.cn; Hao Dai: daihao@sibcb.ac.cn

Supplementary Note 1: Statistic of intercellular gene association network

(1) Construction of statistic

Assuming we want to investigate the association between genes x and y in cell pair k which is composed by cell a_k and b_k. Cell a_k belongs to cell type A and b_k belongs to cell type B. We draw all cell pairs which are constructed by cell type A and B in a scatter diagram (Figure S1). For cell pair k, the x_k is the expression level of gene x in cell a_k, the y_k is the expression level of gene y in cell b_k. Based on the statistical independency in probability theory (eqn. (1-2) in the main text),

we construct our statistic $\rho_{xy}{}^{(k)}$:

$$\rho_{xy}{}^{(k)} = \frac{n_{xy}{}^{(k)}}{n} - \frac{n_x{}^{(k)}}{n} \cdot \frac{n_y{}^{(k)}}{n} \tag{S1}$$

$n_x{}^{(k)}$ and $n_y{}^{(k)}$ are the number of plots in the vertical and horizontal grey boxes. $\frac{n_x{}^{(k)}}{n}$ means the probability of cell type A cell expressing gene x as x_k in all cell pairs. $\frac{n_y{}^{(k)}}{n}$ means the probability of cell type B cell expressing gene y as y_k in all cell pairs. $n_{xy}{}^{(k)}$ is the number of plots in the intersection of the two grey boxes. $\frac{n_{xy}{}^{(k)}}{n}$ means the probability of cell type A cell expressing gene x as x_k and cell type B cell expressing gene y as y_k in all cell pairs.

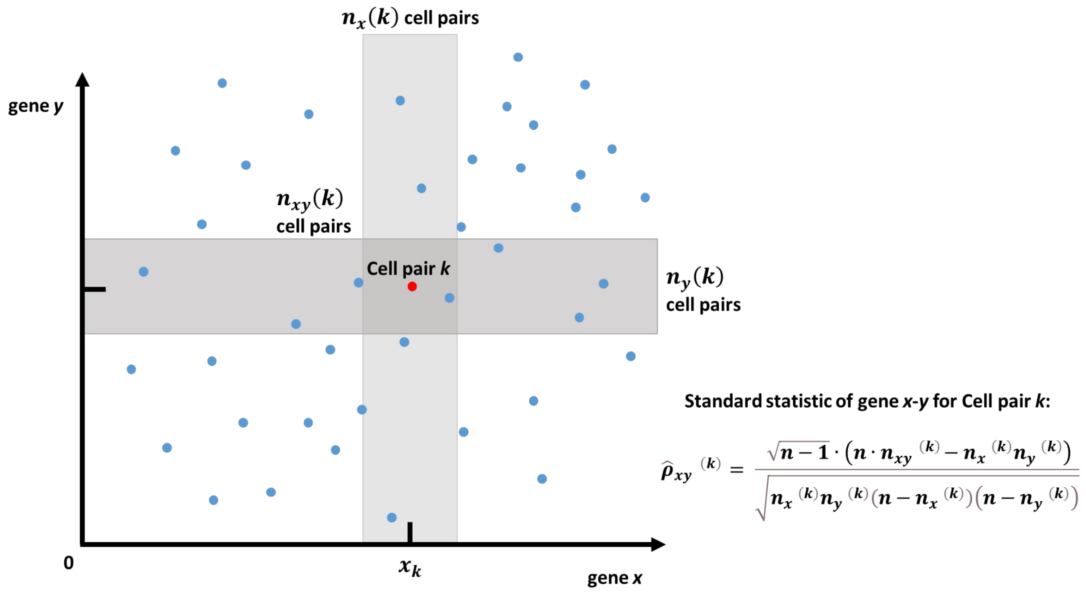

Figure S1. Scatter diagram of the expression values of gene *x*, *y* for cell pair *k*, and illustration of the three boxes for our statistic. Near the red plot k, make the vertical and horizontal grey box to represent the neighborhood of x_k and y_k respectively. The number of plots in the two boxes are $n_x{}^{(k)}$ and $n_y{}^{(k)}$, which are predetermined ($< n$). The intersection of the two boxes is the dark grey box that represents the neighborhood of (x_k, y_k), in which the number of plots is $n_{xy}{}^{(k)}$.

Based on our previous study of the cell-specific gene association network[1]. If the expression of genes x and y are independent of each other in cell pair k, $\rho_{xy}{}^{(k)}$ follows normal distribution and the mean and variance of $\rho_{xy}{}^{(k)}$ are

$$\mu_{xy}{}^{(k)} = 0, \quad \sigma_{xy}{}^{(k)} = \sqrt{\frac{n_x{}^{(k)} n_y{}^{(k)} (n - n_x{}^{(k)})(n - n_y{}^{(k)})}{n^4 (n-1)}} \tag{S2}$$

We normalized $\rho_{xy}{}^{(k)}$ as

$$\hat{\rho}_{xy}{}^{(k)} = \frac{\rho_{xy}{}^{(k)} - \mu_{xy}{}^{(k)}}{\sigma_{xy}{}^{(k)}} = \frac{\sqrt{n-1} \cdot (n \cdot n_{xy}{}^{(k)} - n_x{}^{(k)} n_y{}^{(k)})}{\sqrt{n_x{}^{(k)} n_y{}^{(k)} (n - n_x{}^{(k)})(n - n_y{}^{(k)})}} \tag{S3}$$

Thus, to identify the intercellular gene associations, by using the statistic with our statistical model eqn. (S3), we take the following hypothesis test (one-side test):

H_0 (null hypothesis): genes x of cell a_k and gene y of cell b_k are independent in cell pair k.
H_1 (alternative hypothesis): genes x and y are associated with each other in cell pair k.

If $\hat{\rho}_{xy}{}^{(k)}$ of eqn. (S3) is larger than a significant level (α = 0.05), we will reject the null hypothesis and accept the alternative hypothesis.

(2) Validation of the probability distribution of statistic

To validate the difference in the probability distribution of statistical values between gene pairs with and without correlation, we draw scatter plot of cell pair gene expression and the probability distribution of statistical values for gene pairs based on a true 10X brain spatial RNA-seq dataset (Figure S2). In our model, if only genes x and y are independent, the distribution of $\hat{\rho}_{xy}{}^{(k)}$ follows standard normal distribution and few plots' statistic are larger than the significant level (Figure S2A). If genes x and y are dependent, the distribution of $\hat{\rho}_{xy}{}^{(k)}$ will deviate from the standard normal distribution (Figure S2B-F). The spots which genes x and y are associated have a statistic larger than the significant level and other spots' statistical value tend to be less than 0.

(3) Treatment for the sparsity of spatial transcriptomics

The expression matrix of spatial transcriptome is very sparse, in which most measurements are 0. This leads to a large number of plot pairs located on the coordinate axes in the scatter diagram. As we cannot distinguish whether the zeros come from zero-expressions or just technical problems, it is hard to estimate the neighborhood of the plots on the coordinate axes, and thus the estimation of $\hat{\rho}_{xy}{}^{(k)}$ is inaccurate. So we ignore these plots in calculation, and set $\hat{\rho}_{xy}{}^{(k)} = 0$ in these plots. In other words, we consider the genes with zero expression are not associated with other genes. This treatment to zero expressions can reduce false positive rate in the identification of intercellular gene associations.

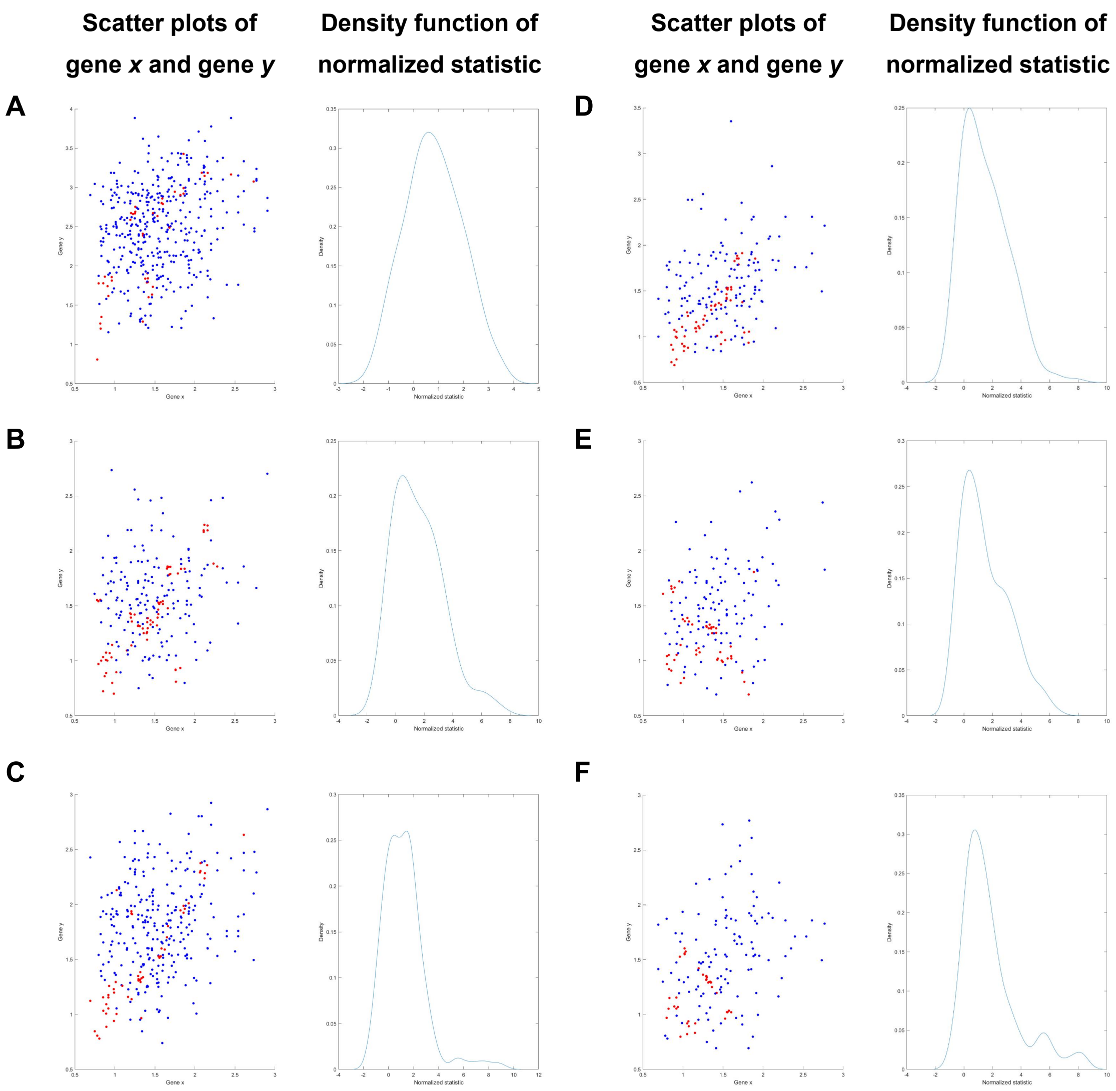

Figure S2. The difference in the probability distribution of statistical values between gene pairs with and without association. A. The case of cell pair without association. It includes scatter plot of cell pair gene expression (left) and the probability distribution of statistical values for gene pairs (right). Its statistical value approximately follows a standard normal distribution. **B-D.** The case of cell pair with positive correlation. In the scatter the red spots represents cell pairs in which the gene pair exhibits correlation. The blue spots represents cell pairs in which the gene pair without correlation. **E-F.** The case of cell pair with negative correlation.

Supplementary Note 2: The usage of IGAN

The input of IGAN is spatial transcriptomic data, including a gene expression matrix and spatial coordinates. First, we used the "Seurat" package to normalize the expression matrix, but without scaling it. Scaling results in cells with the same original count having the same values, which would fail to capture the dynamics of gene expression changes and lead to estimation errors in gene expression probabilities. Next, we clustered all cells to several cell types, and estimated the Euclidean distance between cells to pair up cells in close proximity. As gene expression probability distributions differ among different cell types, we need to group cell pairs. If we did not do this, Equation (2) in the main text would not hold, which requires the consistency of cell types in each group of cell pairs. Therefore, we grouped cell pairs composed of the same cell types, with each group having a similar number of cell pairs. Different numbers of cell pairs would lead to different statistical power in calculations. Additionally, if the number of pairs in each group is too small, the statistic may deviate from a normal distribution, leading to calculation errors. To address this, we spited large groups and merge small ones. In conclusion, the number of pairs in each group should neither be too small nor too large. Figure S3 shows completely different results in different group sizes in a 10X brain dataset. For single-cell spatial RNA-seq data, we recommended grouping them into 5000 pairs, while for spatial bulk RNA-seq data, grouping into 2000 pairs is recommended. Finally, we applied our algorithm to each group to construct a gene association network for each cell pair. After the calculation, we further filtered out gene-gene associations that are not commonly observed. In other words, we removed the results with a discovery frequency lower than 1% of the total cell pairs in the group.

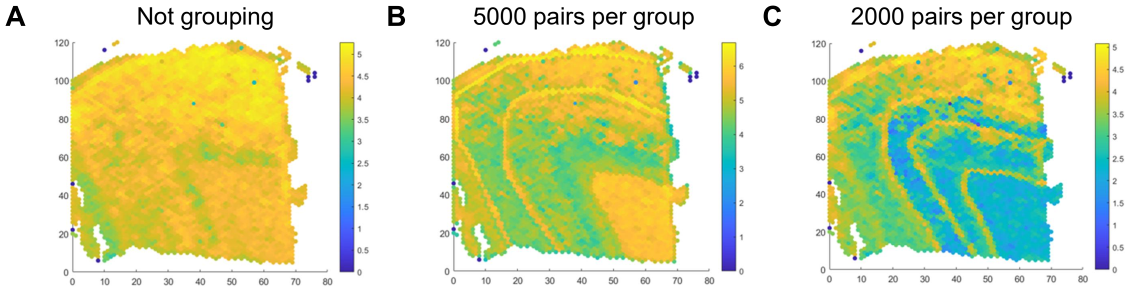

Figure S3. Performance of CCC strength in different group sizes. A. All spots are in the same group. **B.** Each group has around 5000 cell pairs. **C.** Each group has around 2000 cell pairs.

Supplementary Note 3: CCC activity and CCC network of six ligands in the liver cancer dataset

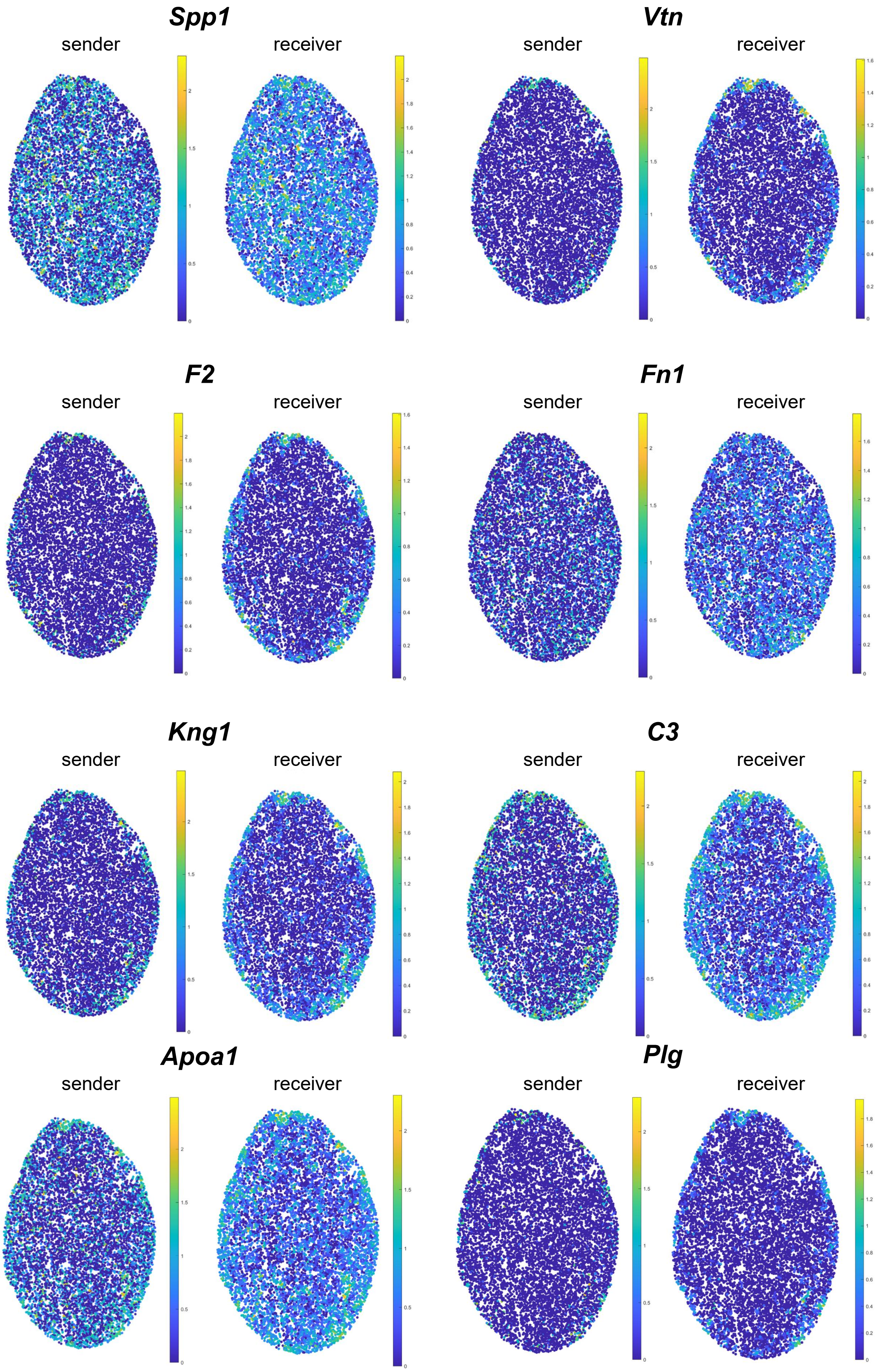

Figure S4. The sending and receiving CCC activity of six ligands in the liver cancer dataset. The graph shows a significant overlap between the highly active signal receiving cells and the signal sending cells. The high activity signal receiving cells cover a larger area than signal sending cells, which indicates that the CCC signals, once emitted from a single cell, can affect multiple neighboring cells.

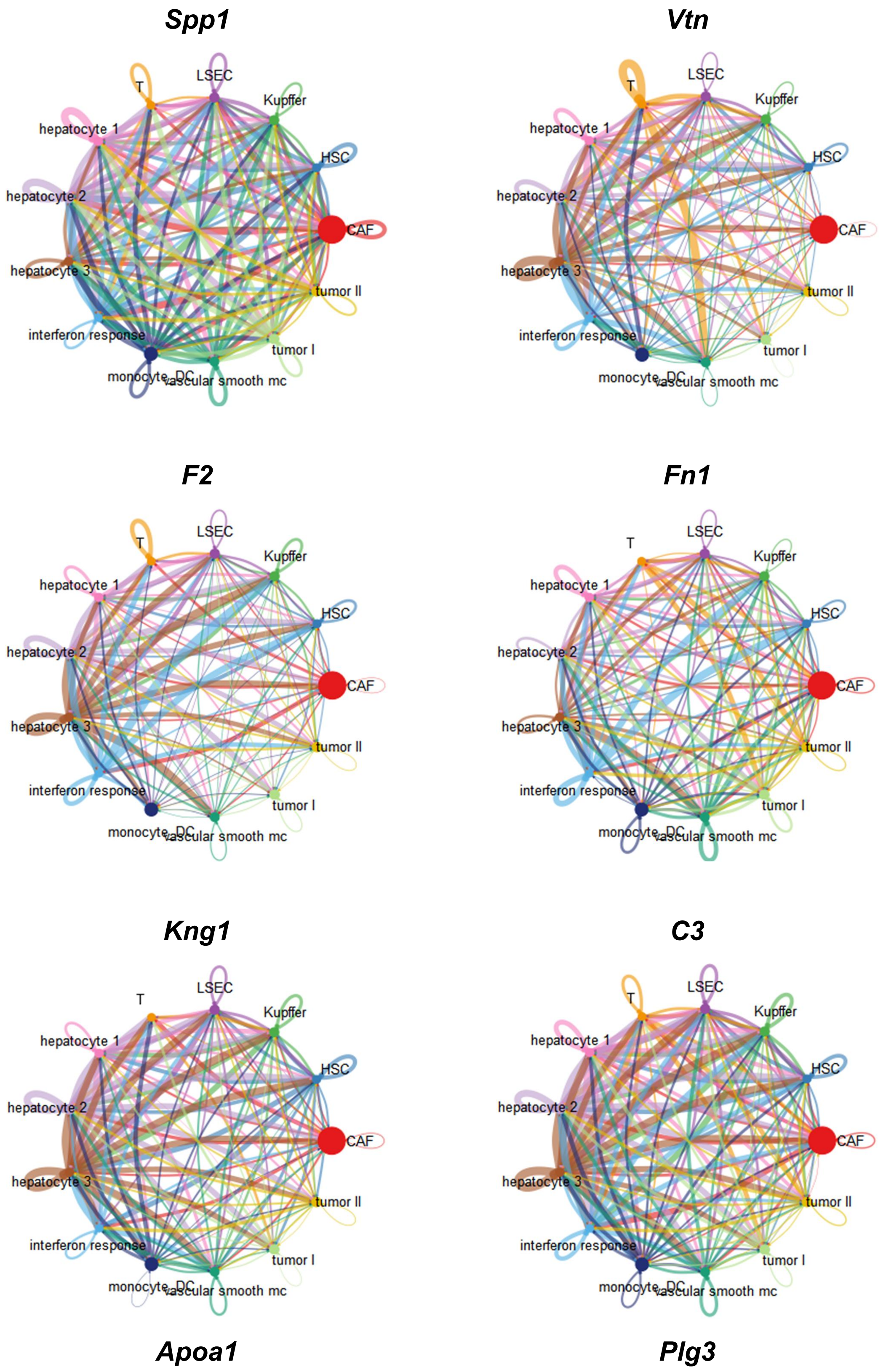
Spp1
Vtn
F2
Fn1
Kng1
C3
Apoa1
Plg3
T
LSEC
Kupffer
HSC
CAF
tumor II
tumor I
vascular smooth mc
monocyte_DC
interferon response
hepatocyte 3
hepatocyte 2
hepatocyte 1

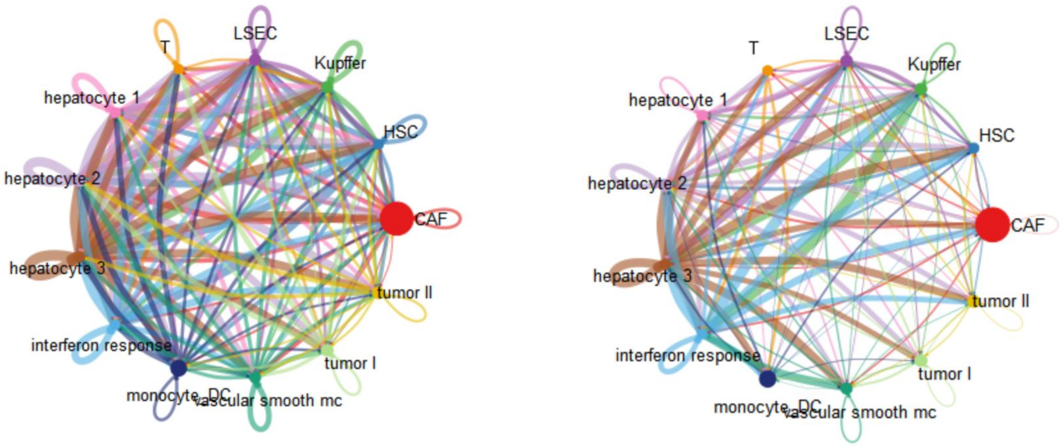

Figure S5. The CCC network of six ligands in the liver cancer dataset. It is drawn by cellchat based on the CCC information produced by IGAN.

Supplementary Note 4: Cell clustering based on CCC information in a mouse spermatogenic tubules dataset

	ES	RS	Myoid	SPC	SPG	Sertoli	Leydig	Endothelial	Macrophage
C0	0.84	0.6	0.11	0.38	0.02	0.05	0	0.12	0.31
C1	0.15	0.37	0.54	0.54	0.63	0.6	0.46	0.63	0.51
C2	0.01	0.02	0.13	0.04	0.19	0.19	0.12	0.12	0.07
C3	0	0	0.14	0.01	0.06	0.05	0.41	0.08	0.07
C4	0	0	0.02	0.01	0.03	0.04	0.01	0.02	0.01
C5	0	0	0.01	0	0.01	0.01	0	0	0.01
C6	0	0	0.01	0	0.01	0.01	0	0	0.01
C7	0	0	0.01	0	0.01	0.01	0	0.01	0
C8	0	0	0.01	0	0.01	0.01	0	0	0
C9	0	0	0.01	0	0.01	0.01	0	0.01	0
C10	0	0	0	0	0.01	0.01	0	0	0
C11	0	0	0	0	0	0	0	0	0
C12	0	0	0	0	0	0	0	0	0
C13	0	0	0	0	0	0.01	0	0	0
C14	0	0	0	0	0	0	0	0	0

Table S1. Clustering performance based on CCC information in a mouse spermatogenic tubules dataset. Columns represent the original cell types, while rows represent the clusters obtained through CCC matrix clustering. The elements in the table indicate the proportion of each original cell type in the CCC cluster. The sum of each row is 1.

Supplementary Note 5: CCC clustering performance and CCC activity of 16 ligands in a cerebellar dataset

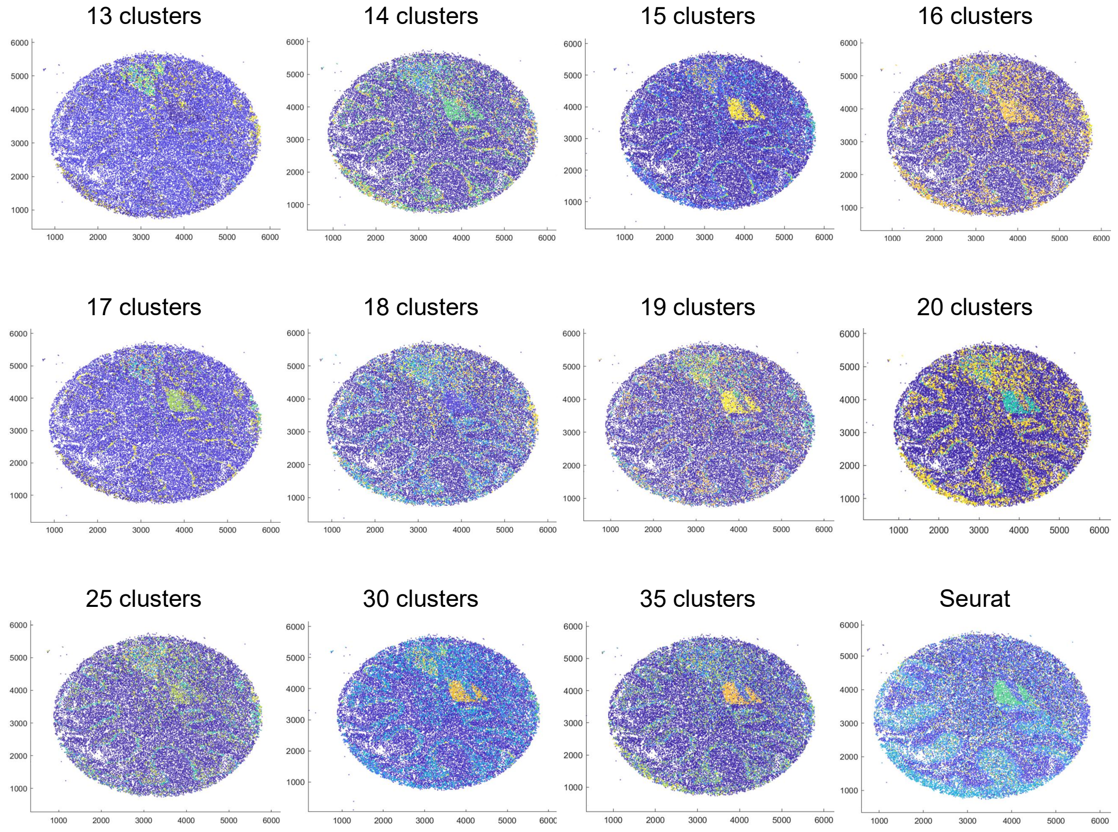

Figure S6. Clustering performance based on CCC information in a cerebellar dataset. The figures display the results with different numbers of categories, as well as the clustering results of the gene expression matrix using Seurat.

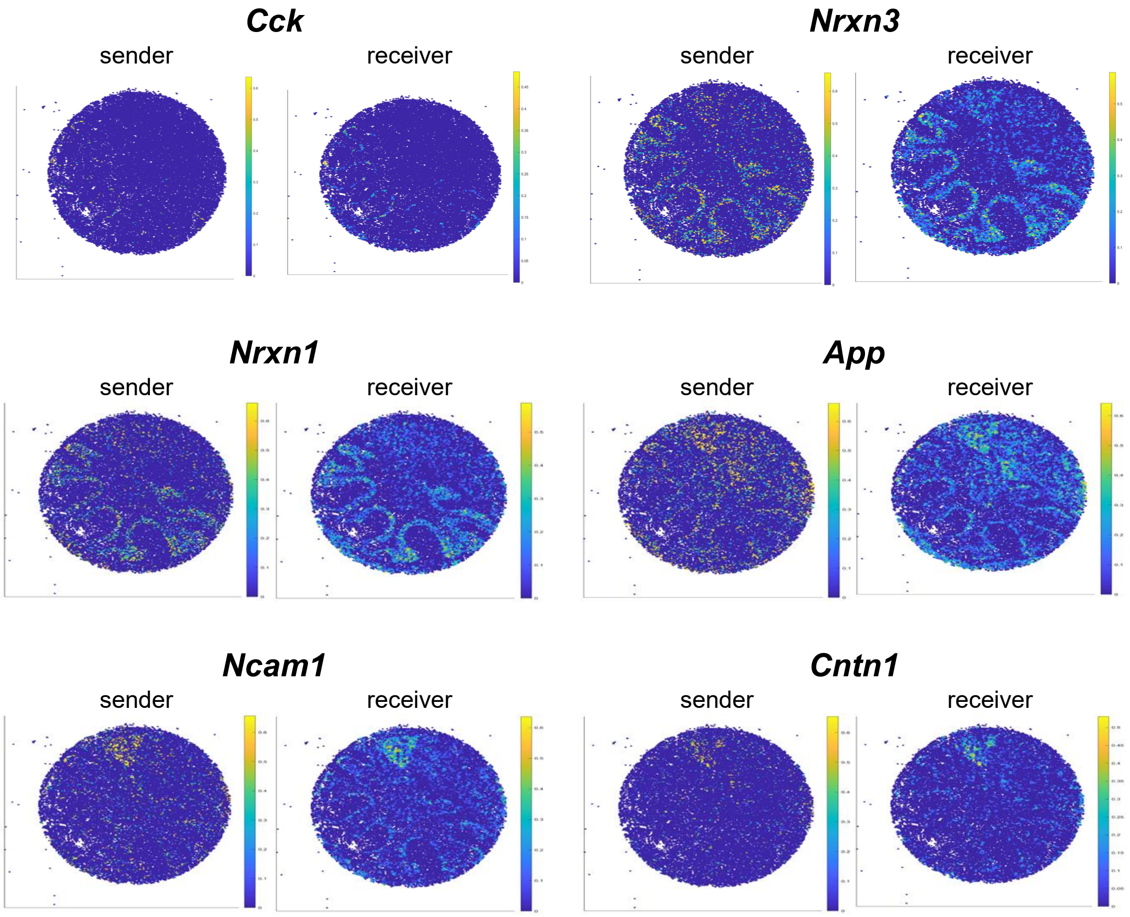

Figure S7. The sending and receiving CCC activity of 16 ligands in the cerebellum dataset.

Supplementary Note 6: CCC activity and CCC network of 23 ligands in the 10X brain dataset

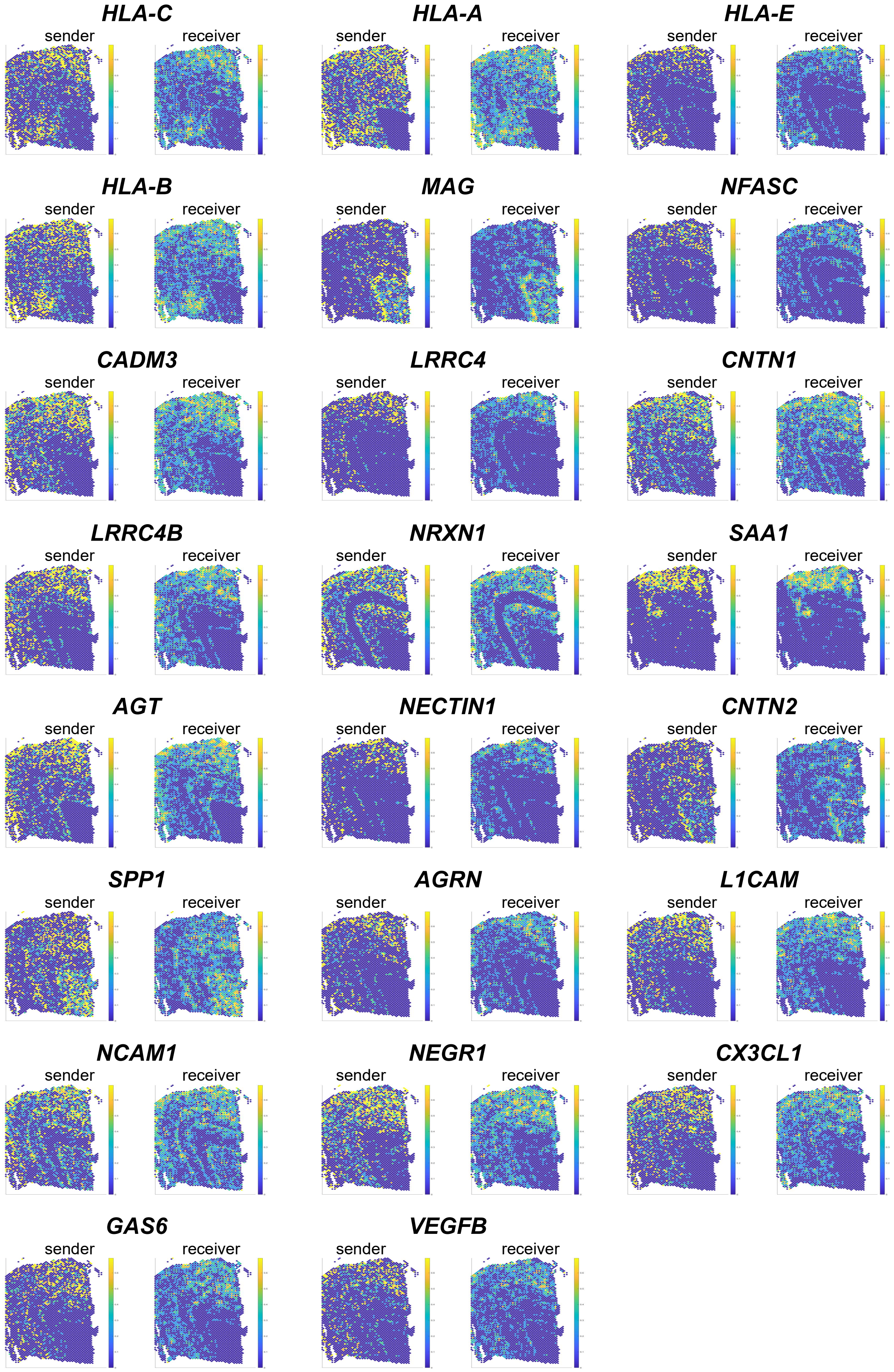

Figure S8. The sending and receiving CCC activity of 23 ligands in the 10X brain dataset.

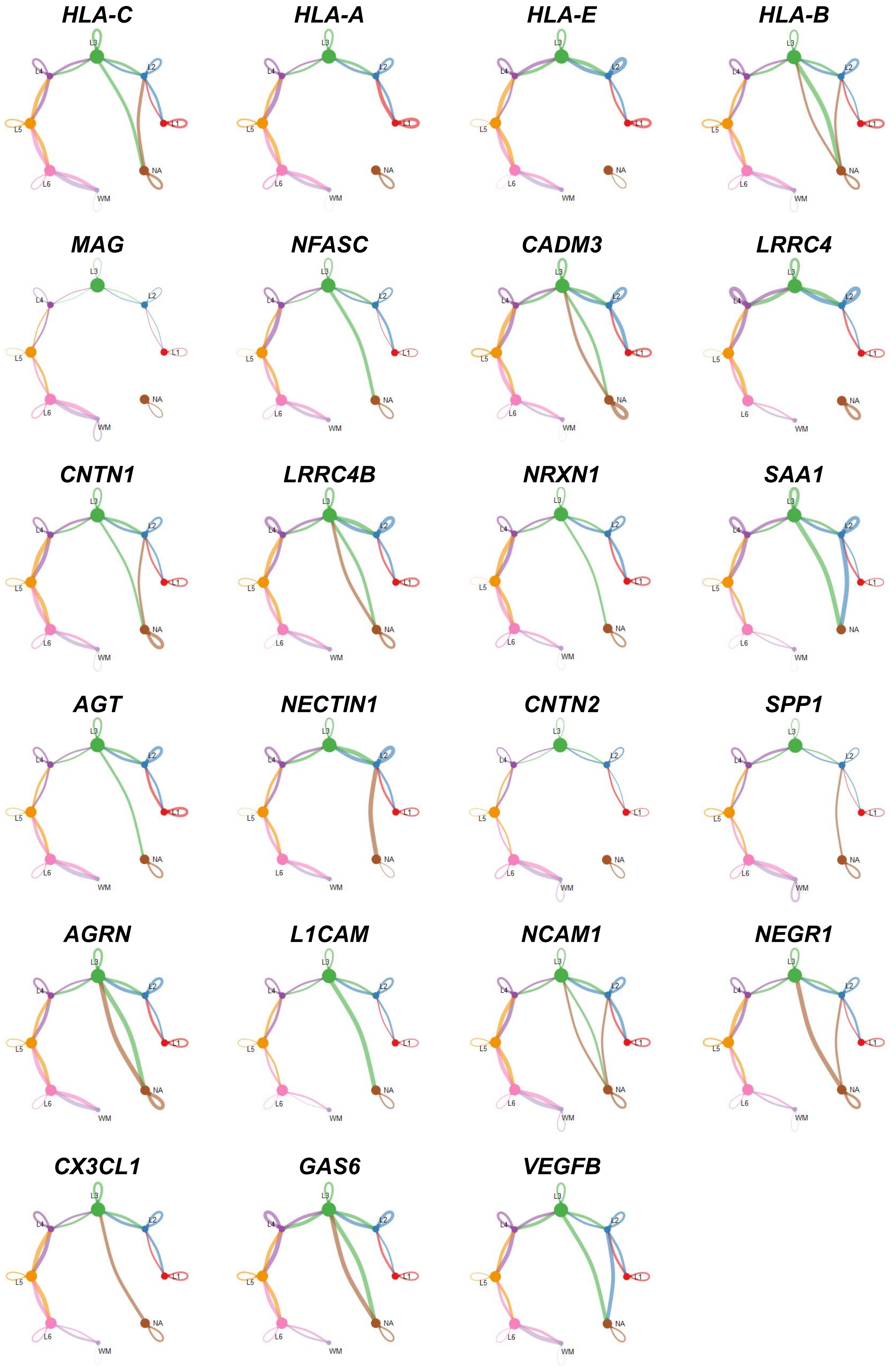

Figure S9. The CCC network of 23 ligands in the 10X brain dataset. It is drawn by cellchat based on the CCC information produced by IGAN.

Supplementary Note 7: Discovery of microenvironment-pattern-specific CCC

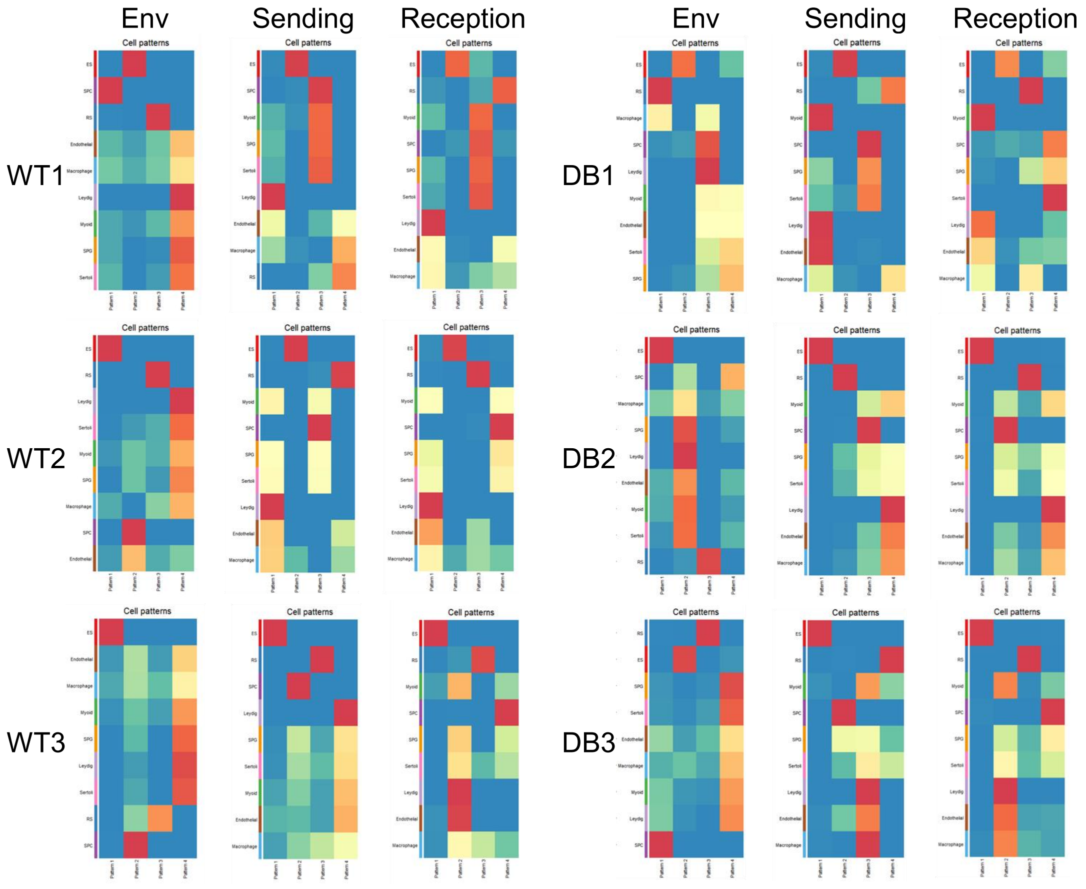

Figure S10. The pattern consistency comparison of the six testes datasets. In each subgraph, the left is microenvironment pattern, the middle is sending CCC pattern, and the right is reception CCC pattern.

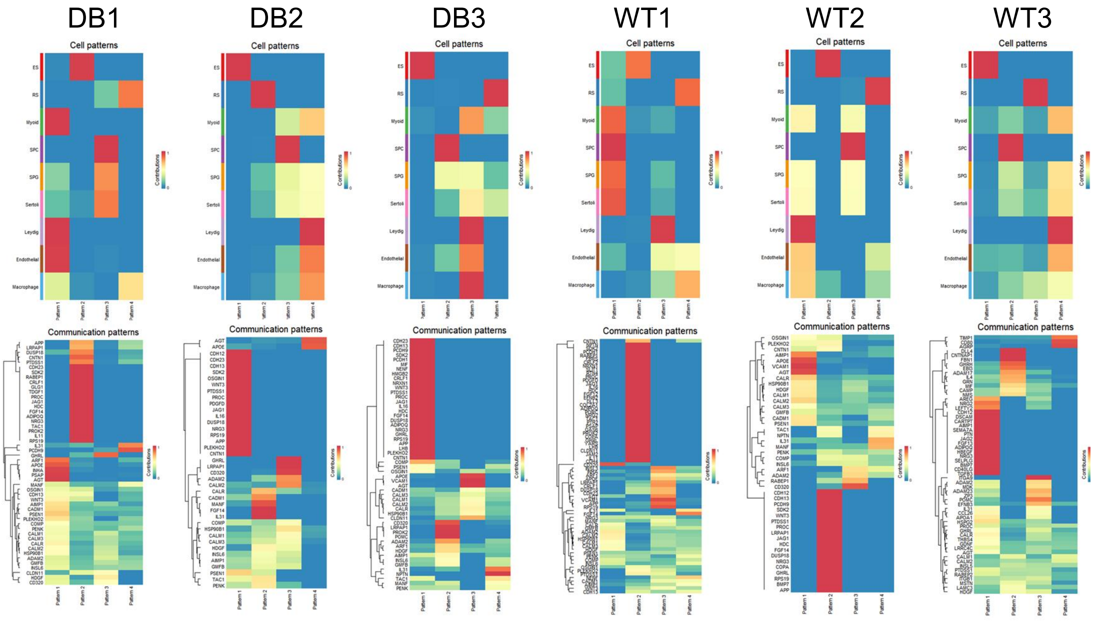

Figure S11. Cell type CCC patterns (top) and contributions of ligands in each CCC pattern (bottom).

Supplementary Note 6: The code of clustering by Seurat

```
obj<- CreateSeuratObject(counts = data)
obj <- Seurat:::NormalizeData(obj)
obj <- FindVariableFeatures(obj, selection.method = "vst", nfeatures = 2000)
all.genes <- rownames(obj)
obj <- ScaleData(obj, features = all.genes)
obj <- RunPCA(obj, features = VariableFeatures(object = obj),npcs = 50)
obj <- FindNeighbors(obj, dims = 1:10)
obj <- FindClusters(obj, resolution = 0.5)
```

Supplementary Note 7: Comparing the CCC (cell-cell communication) activity of sperm cells at different developmental stages.

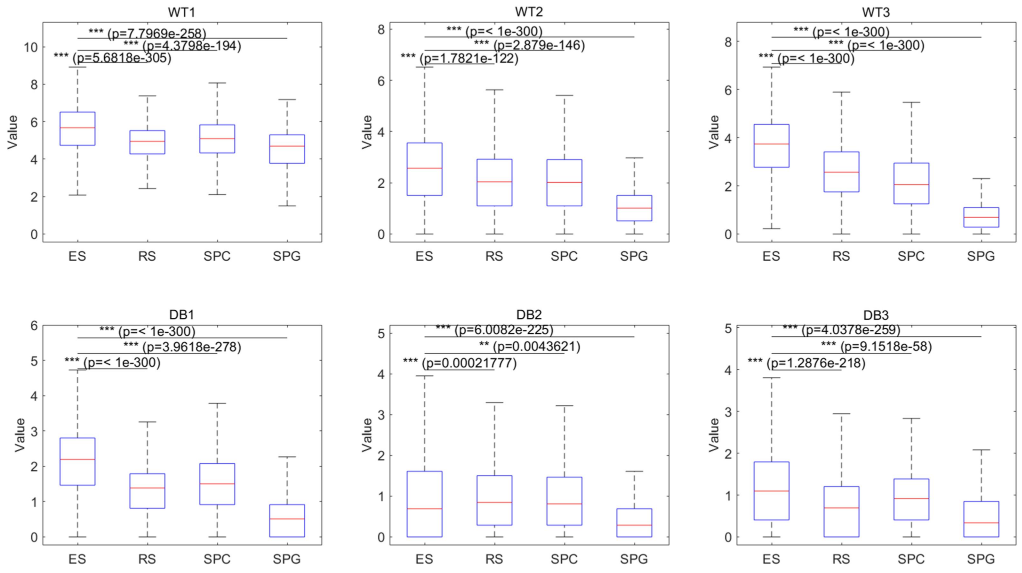

Figure S12. CCC (cell-cell communication) activity comparison of sperm cells in six testes datasets.

Supplementary Note 7: Visualization of ligand and its target gene expression

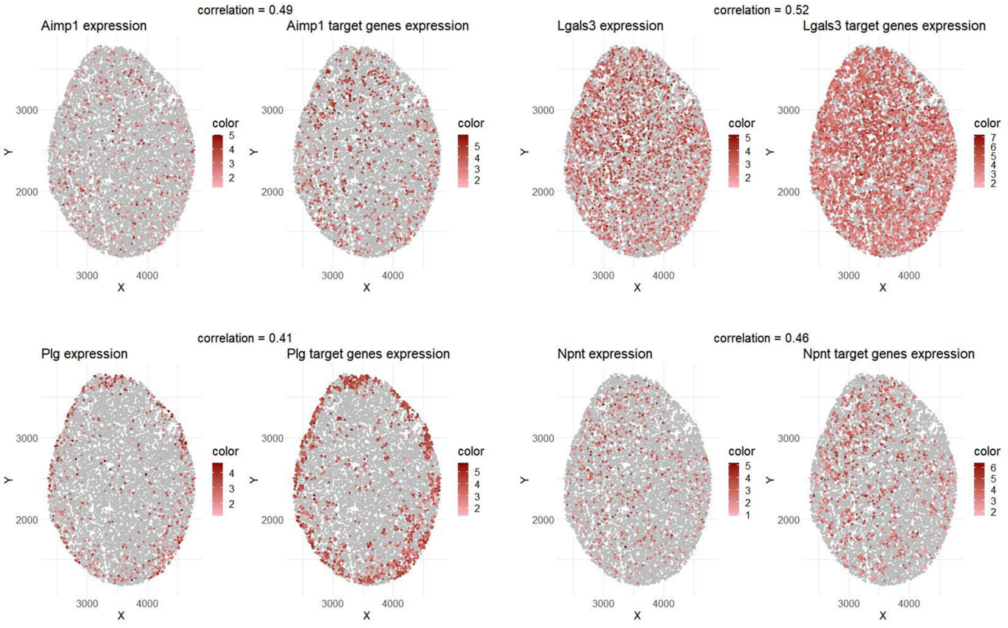

Figure S12. The co-localization of the expression of ligands and the sum of their associated downstream target genes within the correlated cell pairs. The grey points represent cells show no correlation between the ligand and downstream target genes.

DATA AVAILABILITY

All the datasets used in this study are publicly available and accessible:

The slide.seqV2 liver cancer dataset is available at the Broad Institute Single Cell Portal (https://singlecell.broadinstitute.org/single_cell/study/SCP1663), file's name is "SpatialRNA_cropped_slideseq_tumor.rds.zip". It is obtained from KrasG12D/+ Trp53-/- (KP) mouse tumor model and includes 7653 cells and 13 celltypes (CAF, HSC, Kupffer, LSEC, T, hepatocyte 1, hepatocyte 2, hepatocyte 3, interferon response, monocyte_DC, vascular smooth mc, tumor I, tumor II). Every cell has 21902 genes detected.

The slide.seqV2 cerebellum dataset is available at the Broad Institute Single Cell Portal (https://singlecell.broadinstitute.org/single_cell/study/SCP948), files' name are "Cerebellum_BeadLocationsForR.csv" and "Cerebellum_MappedDGEForR.csv". It is collected on the adult mouse cerebellum as the target and includes 39496 cells and 19 cell types(Astrocytes, Bergmann, Candelabrum, Choroid, Endothelial, Ependymal, Fibroblast, Globular, Golgi, Granule, Lugaro, MLI1, MLI2, Macrophages, Microglia, Oligodendrocytes, Polydendrocytes, Purkinje, UBCs). Every cell has 23096 genes detected.

The slide.seqV2 mouse testes datasets are available in the dropbox (https://www.dropbox.com/s/ygzpj0d0oh67br0/Testis_Slideseq_Data.zip?dl=0). It includes 3 WT mouse testes datasets and 3 DB mouse testes datasets. It is obtained from adult male mice of 3–10-month-old and includes 9 celltypes(ES, RS, Myoid, SPC, SPG, Sertoli, Leydig, Endothelial, Macrophage). The seminiferous tubules in these 6 datasets are divided into four stages, representing the four periods of sperm development.

The 10X 151673 DLPFC Human Brain Layers dataset is available through LIBD (http://research.libd.org/spatialLIBD/). It is obtained from the human DLPFC that spans six neuronal layers plus white matter and includes 3639 cells with 24841 genes.

The ligand and target genes' name is produced by NichNet[11] which is available at Zenodo (https://doi.org/10.5281/zenodo.3260758).

REFERENCES

1. Dai H, Li L, Zeng T, Chen L. Cell-specific network constructed by single-cell RNA sequencing data. *Nucleic Acids Research*. 2019; 47(11): e62. doi:10.1093/nar/gkz172
